# Right Ventricular Outflow Tract Obstruction in Monochorionic Twins with Selective Intrauterine Growth Restriction

**DOI:** 10.1155/2012/426825

**Published:** 2012-09-24

**Authors:** S. B. de Haseth, M. C. Haak, A. A. W. Roest, M. E. B. Rijlaarsdam, D. Oepkes, E. Lopriore

**Affiliations:** ^1^Division of Fetal Medicine, Department of Obstetrics, Leiden University Medical Center, 2300 RC Leiden, The Netherlands; ^2^Division of Pediatric Cardiology, Department of Pediatrics, Leiden University Medical Center, 2300 RC Leiden, The Netherlands; ^3^Division of Neonatology, Department of Pediatrics, Leiden University Medical Center, 2300 RC Leiden, The Netherlands

## Abstract

Monochorionic twin pregnancies are at increased risk of perinatal mortality and morbidity due to twin-twin transfusion syndrome (TTTS), selective intrauterine growth restriction (sIUGR), and higher incidence of congenital heart malformations. The incidence of right ventricular outflow tract obstruction (RVOTO) in recipients with TTTS is known to be higher than in the general population. There is limited data on the risk of RVOTO in monochorionic twins with sIUGR. We report a case of RVOTO in the larger twin in a monochorionic twin pregnancy with sIUGR, treated successfully with balloon dilatation after birth.

## 1. Introduction

Monochorionic pregnancies are at increased risk of major complications, including twin-twin transfusion syndrome (TTTS), twin anemia polycythemia sequence (TAPS), and selective intrauterine growth restriction (sIUGR) [1]. In addition, monochorionic twins have an increased incidence of congenital malformations, mainly heart malformations and central nervous system defects. In TTTS, the recipient twin can develop cardiac hypertrophy, tricuspid regurgitation, and right ventricular outflow tract obstruction (RVOTO), in an initially normal developed heart [2, 3]. The etiology of RVOTO in recipients with TTTS is not clear and may be associated with increased preload (hypervolemia) and/or afterload (increased levels of endothelin-1) [4].

 Whether the risk of RVOTO is also increased in other subgroups of monochorionic pregnancies besides TTTS is not well known. We report a monochorionic twin pregnancy with sIUGR in which RVOTO was detected in the larger twin in the 2nd trimester of pregnancy and discuss possible causative mechanisms.

## 2. Case Report

**Figure 1:**
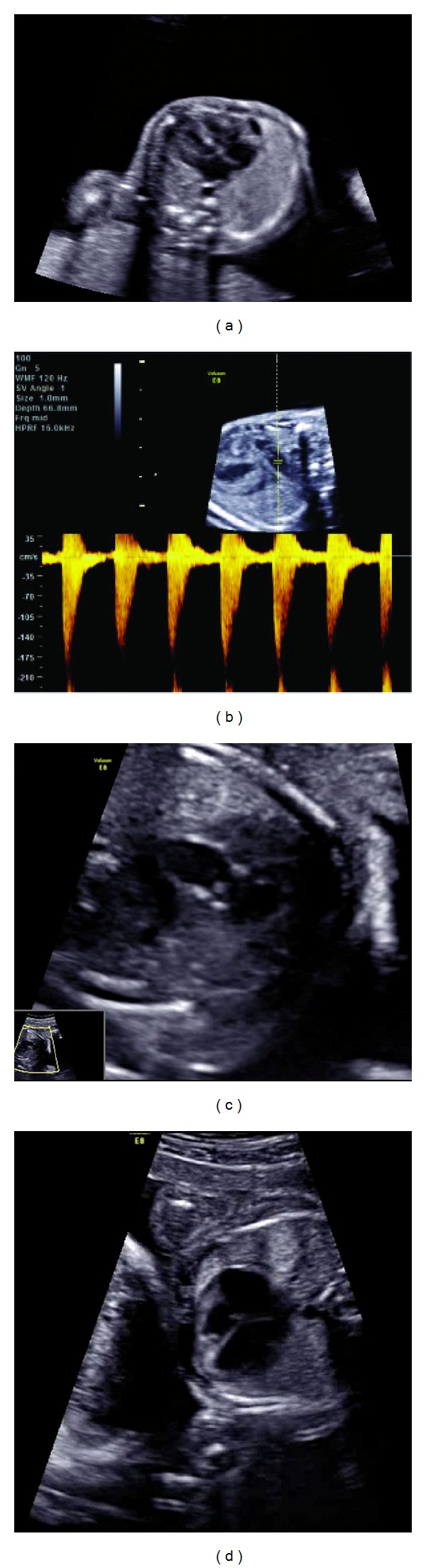
(a) Ultrasound examination at 21 weeks GA shows a four chamber view with a very mild right ventricle hypertrophy and mildly pericardial effusion. (b) Ultrasound examination at 21 weeks GA shows an elevated peak systolic velocity across the pulmonary valve of 1.75 m/s. (c) Ultrasound examination at 21 weeks GA shows the pulmonary valve with a mild post stenotic dilatation. Valve cusps and pulmonary wall were echogenic and thickened. (d) Ultrasound examination at 28 weeks GA shows RV remains adequate, mild hypertrophy.

A 35-year-old woman, gravida 3, para 2, was referred to our center at 15 + 0 weeks of gestation for suspected TTTS in a monochorionic-diamniotic pregnancy [5]. Our center serves as the national referral center for fetoscopic laser surgery for TTTS in the Netherlands. Ultrasound examination showed a normal amount of amniotic fluid (deepest vertical pocket (DVP) 4 cm) in one twin (twin A) and a reduced amount of amniotic fluid (DVP 2 cm) in the other twin (twin B). Both twins had normal bladder filling. Diagnostic criteria for TTTS were thus not fulfilled. The estimated fetal weight (EFW) of fetus A was p50, biometry of fetus B revealed growth <p10. Doppler ultrasound revealed intermittent absent end-diastolic flow in umbilical artery in the smallest twin. These findings suggest a type III selective intrauterine growth restriction (sIUGR) [6]. After counseling, the parents opted for expectant management and no interventions were performed. Ultrasound examination was repeated weekly. At 17 + 1 weeks of gestation, mild pericardial effusion was seen in the larger twin, in a furthermore normal heart. At 19 + 1 weeks, the pulmonary valve in the larger twin had a normal diameter for gestational age, but they showed echogenic valve cusps and a mildly elevated peak systolic velocity across the pulmonary valve (1 m/s). Two weeks later, at 21 + 3 weeks of gestation, the abnormalities progressed with myocardial hypertrophy of the right ventricle and a peak systolic velocity across the pulmonary valve of 1.75 m/s. The antegrade flow was combined with mild insufficiency at the beginning of the diastole (Figure 1). In addition, tricuspid regurgitation was present and the ductus arteriosus showed retrograde flow. The right ventricle had a normal size. The myocardial hypertrophy progressed during the pregnancy, the other cardiac abnormalities remained stable until birth. 

The patient was admitted to our hospital at 34 + 3 weeks of gestational age for induction of labor. Two male infants were born vaginally with a birth weight of 2629 grams (twin A) and 1850 grams (<p10) (twin B). The Apgar scores in twin A were 8, 9, 9 and in twin B and 9, 10, 10 at 1, 5, and 10 minutes, respectively. 

**Figure 2:**
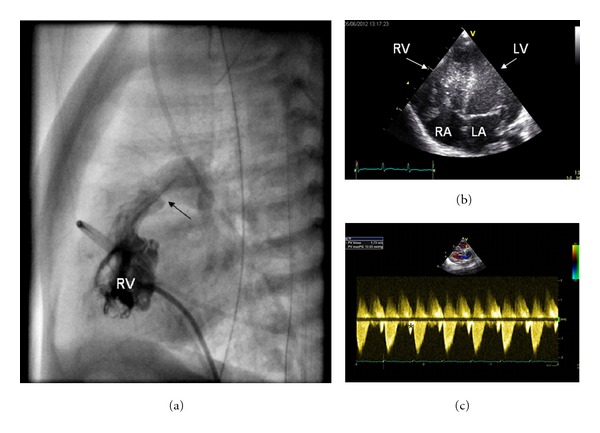
(a) Depicts the stenosis of the pulmonary valve during contrast injection into the right ventricle (RV) obtained during cardiac catheterization. Note the very small opening of the pulmonary valve (arrow). (b) Shows an apical 4-chamber view obtained with echocardiography. Note the echogenic hypertrophied trabeculae within the cavum of the RV. (c) Shows the continuous wave Doppler tracing along the main pulmonary artery with a peak gradient of 12 mmHg. Furthermore, the end diastolic forward flow over the pulmonary valve is clearly depicted (*) as a sign of restrictive RV filling. LA: left atrium, LV: left ventricle, RA: right atrium, RV: right ventricle.

Echocardiography of the larger twin after birth showed severe pulmonary valve stenosis with tricuspidvalve insufficiency and severe hypertrophy of the right ventricle. Treatment with i.v. prostaglandins was started on day 1. Five days after delivery, a balloon valvuloplasty was performed successfully. The neonate recovered quickly from the procedure, and was weaned from the prostaglandins and the saturation remained stable. Postinterventional echocardiography revealed a transpulmonary gradient of 12 mmHg with trivial pulmonary valve insufficiency. Moderate tricuspid valve insufficiency persisted, as well as hypertrophy of the right ventricle with end-diastolic forward flow over the pulmonary valve, as a sign of restrictive ventricular filling (Figure 2).

Both twins were discharged from the hospital in good clinical condition on day 11. 

**Figure 3:**
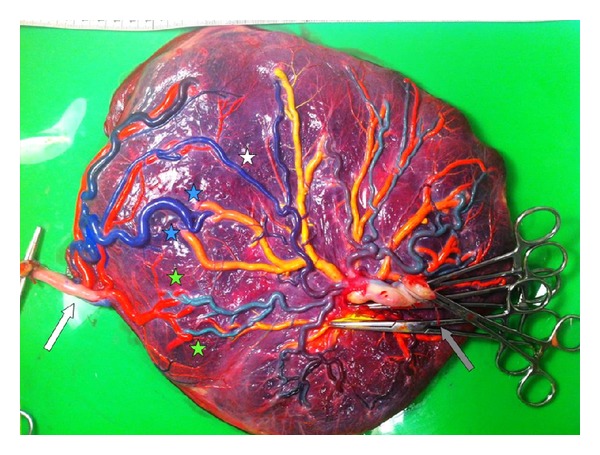
Monochorionic placenta after color dye injection (blue and green colors for arteries, red and yellow colors for veins) showing typical features of unequal sharing. Twin B has a velamentous cord insertion (white arrow) and a small placental territory (placental share on the left side of the picture). Twin A has a paracentral cord insertion (grey arrow) and a larger placental territory (right side of the picture). The white star indicates a large arterio-arterial anastomosis, the blue stars and green stars indicate several arterio-venous en veno-arterial anastomoses, respectively.

The placenta showed evident signs of unequal sharing with a velamentous insertion for twin B. After color dye injection, 1 large arterio-arterial (A-A) anastomosis and several arterio-venous (A-V) and veno-arterial (V-A) anastomoses were detected (Figure 3). 

## 3. Comment

RVOTO has been reported to occur more frequently in recipient twins with TTTS, with an incidence ranging from 4 to 9% [3, 4]. This is more than 10 times higher than in the general population [7]. Our report shows, for the first time, that RVOTO can also occur in monochorionic twins complicated with sIUGR. We speculate that a common pathway could play a role in the development of RVOTO in both monochorionic twins with TTTS as in monochorionic twins with sIUGR. 

The pathogenesis of RVOTO in recipients with TTTS is not clear. RVOTO has been linked to increased preload due to volume overload following fetofetal transfusion through placental vascular anastomoses as well as to increased afterload due to high levels of vasoconstrictive hormones such as endothelin-1 [4]. It is hypothesized that increase preload and/or increased afterload in recipient twins may both lead to fetal systemic hypertension and the development of hypertrophic cardiomyopathy and eventually RVOTO [2, 4]. As RVOTO has been linked to hemodynamic disturbances in fetal life, RVOTO appears to be partly an “acquired” congenital heart defect. Muñoz-Abellana et al. suggested that cardiac abnormalities in the larger twin in monochorionic twins with sIUGR may be caused by a hyperdynamic state due to the disproportion of the placental territory in combination with a large A-A anastomosis [8]. The development of RVOTO in monochorionic twins with sIUGR may also be linked to hemodynamic imbalances related to the presence of placental vascular anastomoses. The placenta in our case of sIUGR without TTTS showed a large A-A and several A-V anastomoses. The smaller twin showed abnormal flow velocity patterns in the umbilical artery, including intermittent reversed end-diastolic flow. We hypothesize that a contributing factor in the abnormal valve development in RVOTO in these cases could be the occurrence of short but frequent volume shifts during the periods with reversed end-diastolic flow of the smaller twin. A large A-A anastomosis, as present in this case, will propagate pre- and afterload differences directly to the larger cotwin. The exact mechanism and necessary conditions to develop RVOTO are, however, not yet elucidated.

In conclusion, detecting RVOTO antenatally is important as RVOTO may be progressive and require urgent treatment with pulmonary balloon valvuloplasty or surgery after birth. Since RVOTO may evolve until delivery, serial fetal echocardiograms and careful monitoring are mandatory. Perinatologists should be aware that RVOTO may not only occur more frequently in recipient twins with TTTS, but can also occur in monochorionic twins with sIUGR. Whether the incidence of RVOTO in monochorionic twins with sIUGR is increased compared to the general population requires further study. 

## Abbreviations

A-A: Arterio-arterial
A-V: Arterio-venous
CHD: Congenital heart disease
GA: Gestational age
MC: Monochorionic
RV: Right ventricle
RVOTO: Right ventricular outflow tract obstruction
sIUGR: Selective intrauterine growth restriction
TTTS: Twin-to-twin transfusion syndrome
V-A: Venous-arterial.
